# Development of Online Child Support Activities by Older Adults; An Action Research During the COVID-19 Pandemic

**DOI:** 10.1093/geroni/igab046.3062

**Published:** 2021-12-17

**Authors:** Mai Takase, Ryogo Ogino, Ryoichi Nitanai, Jun Goto

**Affiliations:** 1 The University of Tokyo, Bunkyo-ku, Tokyo, Tokyo, Japan; 2 Saga University, Saga, Saga, Japan; 3 the University of Tokyo, Tokyo, Tokyo, Japan; 4 Tokai University, Hiratsuka, Kanagawa, Japan

## Abstract

Japanese communities have been attempting a novel type of childcare support, wherein community-dwelling older adults form a specialized group (support group) that aims to provide child support activities. Before the COVID-19 pandemic, the group gathered children and mothers in community spaces and conducted events. However, on-site support had to be halted due to the pandemic. In this study, we report a case of action research aimed at shifting the activities online. First, a suitable online support plan was explored by hosting several discussions with child-rearing mothers. A questionnaire survey was then conducted to determine the most-sought intervention contents (N=19). Finally, based on the results, an intervention was conducted. As a result of the discussions, the hosting of online programs was set as the main goal. Out of the ten activities studied, the three most popular activities were programming (n=17), English conversation (n=16), and science workshop (n=15). Based on the results, an online science workshop that built a Bottleium, a small aquarium using a bottle, was hosted. Eight children participated in the event. A post-activity survey revealed that all participants attended the online activity for the first time, and the parents were happy to have joined the activity that entertained their child during the quarantine period. Furthermore, focus group interviews were conducted with the support group; they reported being satisfied with the outcome and recognized the importance of their role as member of support group. The results suggest the positive effect of this project on both older adults and the children.

